# Imaging Bell-type nonlocal behavior

**DOI:** 10.1126/sciadv.aaw2563

**Published:** 2019-07-12

**Authors:** Paul-Antoine Moreau, Ermes Toninelli, Thomas Gregory, Reuben S. Aspden, Peter A. Morris, Miles J. Padgett

**Affiliations:** School of Physics and Astronomy, University of Glasgow, Glasgow G12 8QQ, UK.

## Abstract

The violation of a Bell inequality not only attests to the nonclassical nature of a system but also holds a very unique status within the quantum world. The amount by which the inequality is violated often provides a good benchmark on how a quantum protocol will perform. Acquiring images of such a fundamental quantum effect is a demonstration that images can capture and exploit the essence of the quantum world. Here, we report an experiment demonstrating the violation of a Bell inequality within observed images. It is based on acquiring full-field coincidence images of a phase object probed by photons from an entangled pair source. The image exhibits a violation of a Bell inequality with *S* = 2.44 ± 0.04. This result both opens the way to new quantum imaging schemes based on the violation of a Bell inequality and suggests promise for quantum information schemes based on spatial variables.

## Teaser

We report the violation of a Bell inequality within full-field coincidence images of a phase object probed by entangled photons.

## INTRODUCTION

Quantum entanglement makes a key distinction between the classical and the quantum world. The debate over the interpretation of this entanglement remained center stage for much of the 20th century. That an interpretation based on hidden variables could be ruled out on the basis of experimental observation is the essence of the Bell inequality, and following the seminal works of Freedman and Clauser (*1*) and Aspect *et al.* (*2*–*4*), many groups worldwide have used nonlocal correlations between pairs of photons to show the violation of this type of inequality. Notably, recent improvements in the scheme design and component performance have allowed simultaneous closing of the various loopholes present in earlier demonstrations (*5*–*7*).

The violation of a Bell inequality is a fundamental manifestation of a quantum system. Not only does it attest to the quantum spookiness of the behavior of a system, but it also benchmarks the performance of these systems when involved in certain quantum protocols. For example, certain quantum protocols require Bell-type nonlocal behaviors to be performed such as device-independent protocols (*8*–*10*). One quantum technology that is currently of interest is quantum imaging that attempts to use the quantum behavior of light to perform new types of imaging that are capable of surpassing the limits of classical methods. As such, acquiring images of one of the most fundamental quantum effects is a demonstration that images can be exploited to access the full range of possibilities allowed in the quantum world.

The violation of Bell inequalities, ruling against hidden variable interpretations of quantum mechanics, has usually relied on the sequential measurement of correlation rates as a function of analyzer settings (e.g., the relative angles of linear polarizers) acting on the two photons separated in space from each other. A violation of the Bell inequality dictates that the correlation rate depends not on the angle of either polarizer alone but on the combination of both. The nonlocal nature of this correlation gives rise to the term “spooky action at a distance.” Measuring polarization is convenient, but tests of entanglement have been performed with other variables too (*11*–*14*), and although the original Bell inequality was applied to variables within a two-dimensional Hilbert space, a similar logic can be followed to design tests in higher-dimensional state spaces (*15*).

In understanding our present work, it is important to consider two of the other high-dimensional domains in which entanglement can be explored. The first high-dimensional domain is that rather than analyzing the polarization and, hence, the spin angular momentum of the photons, an alternative is to measure the orbital angular momentum (OAM) of the photons. The OAM of ℓℏ per photon arises from the helical phase structure of the beam described as exp(*i*ℓϕ) (*16*, *17*). Although early experiments on this OAM concentrated on observing its mechanical manifestations (*18*), later work examined the correlations of OAM between photons produced by parametric down-conversion showing entanglement (*19*) and, subsequently, a violation of a Bell-type inequality in two-dimensional (*20*) and higher-dimensional (*21*) OAM subspaces. The second high-dimensional domain relates to Einstein et al. (*22*), who famously expressed their concerns on the completeness of quantum mechanics through the EPR (Einstein-Podolsky-Rosen) paradox. This paradox concerns the correlations between the position and momentum correlations that might be expected to occur between the two entangled particles. For photon pairs produced by parametric down-conversion, both spatial correlations and momentum anticorrelations can be observed in the image plane and far-field of the source, respectively. These correlations are the basis of quantum ghost imaging (*23*, *24*), where one of the two down-converted beams is directed to the object, with a single pixel (nonspatially resolving) detector, collecting the interacting light, and the other beam is directed to an imaging detector. The data from neither detector alone give an image of the object, but a summation of the correlations between the nonimaging and imaging detectors does. If the object and the spatially resolving detector are in the image planes of the source, then the image is upright with respect to the object; if the object and the detector are in the far-field of the source, then the image is inverted with respect to the object. The upright or inverted nature of the image arises from position correlation and momentum anticorrelation, respectively, and can be considered to be an image-based manifestation of the EPR paradox (*25*). In a different context, the demonstration of an EPR paradox in imaging has been performed in a series of experiments (*26*, *27*, *28*, *29*) where cameras were used to image the position and momentum of entangled pairs of photons. This eventually led to the demonstration of an EPR paradox within single frames of a detector array (*30*). The question that this present work seeks to demonstrate is what kind of imaging process could reveal a Bell inequality?

The key to measurements in the polarization experiments is to recognize that the orientation of the linear polarizer analyzer is actually a measurement of the phase difference between a superposition of the right- and left-hand circular polarization states (i.e., the spin angular momentum states). A superposition of right- and left-hand OAMs (ℓ = ± 1) has a π-phase step across the diameter of the beam, where the phase difference between the two OAM states sets the orientation of the step. One notes that a circle contains all possible edge orientations, and hence, as an object, a circle is equivalent to a full rotation of the polarizer in the polarization case. This has been exploited in a previous demonstration where the nonlocal detection of phase steps showed an edge enhancement dependent on the edge orientation that was indicative of a violation of the Bell inequality (*31*). However, that realization did not rely on imaging because the data were acquired sequentially by scanning an object within a spatially single-mode optical setup. The data from this ensemble of measurements were eventually recombined to be presented in the form of an image. By contrast, in this present work, we use a full-field imaging configuration where a phase-edge filter was placed nonlocally with respect to a circular phase object to give a ghost image, the intensity features of which reveal the anticipated violation of a Bell-type inequality for OAM. This experiment requires no scanning of any kind, relying on the use of a quantum state that can exhibit correlations in the OAM space or in the direct Cartesian space. It illustrates that Bell-type nonlocal behavior can be demonstrated within a full-field quantum imaging protocol. Because we do not close all the various loopholes, our demonstration cannot be interpreted as another absolute demonstration that the world is behaving in a nonlocal way. However, these loopholes are not fundamentally associated with the experimental paradigm presented here and could be, in principle, closed with technically more advanced detectors and phase-image displays. In addition, as we will discuss below, by making only a few physically reasonable assumptions about the source involved in the demonstration, our results can be interpreted as the first experimental demonstration that an imaging protocol can be used to reveal the Bell-type–violating behavior of a quantum system. Reciprocally, our results do show that Bell-type nonlocal behavior can be harnessed to perform special types of imaging that could not be performed with a conventional classical source.

## RESULTS

### Experimental setup and principle of the demonstration

**Fig. 1:**
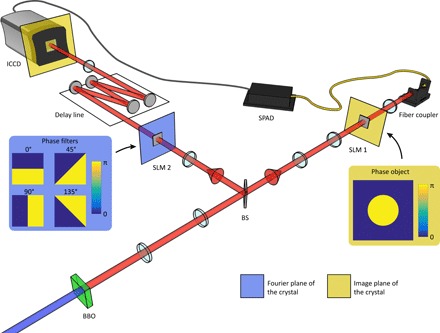
Imaging setup to perform a Bell inequality test in images. A BBO crystal pumped by an ultraviolet laser is used as a source of entangled photon pairs. The two photons are separated on a beam splitter (BS). An intensified camera triggered by a SPAD is used to acquire ghost images of a phase object placed on the path of the first photon and nonlocally filtered by four different spatial filters that can be displayed on an SLM (SLM 2) placed in the other arm. By being triggered by the SPAD, the camera acquires coincidence images that can be used to perform a Bell test.

Our experimental system, shown in Fig. 1, consists of a β-Barium Borate (BBO) crystal pumped by a quasi-continuous laser at 355 nm, thereby generating spatially entangled pairs of photons at 710 nm through the process of spontaneous parametric down-conversion (SPDC). The two photons are separated on a beam splitter and propagate into two distinct optical systems (arms). The first photon is reflected off a spatial light modulator (SLM) placed in an image plane of the crystal and displaying a phase object before being collected inside a single-mode fiber (SMF) and is subsequently detected by a single-photon avalanche diode (SPAD). The second photon, traveling through the other arm, is reflected off an SLM placed in a Fourier plane of the crystal (equivalent to the Fourier plane of the object) and displays a spatial π-phase step filter. The photon then propagates through a ~20-m-long, image preserving, delay line before being eventually detected by an intensified charge-coupled device (ICCD) camera. The ICCD camera is triggered conditionally on the detection of a photon by the SPAD placed in the first arm. This delay line ensures that the images obtained from the ICCD camera are coincidence images with respect to the SPAD detection. The presence of the delay line in the second arm compensates for the trigger delays of the camera and ensures that the second photon is incident on the camera during the 4-ns gate time of the image intensifier.

We have used such a triggering mechanism to implement a quantum illumination protocol and acquire images with fewer than one photon per pixel (*32*) and in the context of phase and amplitude imaging (*33*). We also used a similar setup to test the experimental limits of ghost imaging and ghost diffraction (*34*, *35*). In the presently reported work, our scheme uses phase imaging to give edge enhancement through spatial filtering. Here, the object (a circular phase step) and the filter (a straight-edged phase step) are placed nonlocally within separated optical arms and are probed by two spatially separated but entangled photons. The resulting edge-enhanced image of the circle is a result of the nonlocal interference between the object and the spatial filter probed by the two-photon wave function. However, simply obtaining an edge-enhanced image in these circumstances is not in itself a proof of the nonlocal character of the two photons’ behavior in that it can potentially be reproduced by classical means as in the context of ghost imaging (*24*). One can nonetheless produce images that cannot be reproduced by classical means through the demonstration of the violation of a Bell inequality.

An understanding that a Bell inequality can be violated by the implementation presented on Fig. 1 can be drawn from the realization that a π-phase step has both ℓ = 1 and ℓ = −1 contributions when expressed in an OAM basis. A π-phase step can, in fact, be represented as the linear superposition of ℓ = −1 and ℓ = 1 and, thus, can be represented on a Bloch-Poincaré sphere (*36*) describing a two-dimensional OAM basis. In this context, the phase difference θ between the two modes ℓ = −1 and ℓ = 1 determines the orientation angle θ of the π-phase step in the two-dimensional transverse plane. One can therefore use these phase steps as filters to perform measurements in this particular two-dimensional OAM space (*20*). Projected purely into such a space, the two-photon wave functions can be written in the following way

$$|\psi \rangle ={|-1\rangle }_{1}{|1\rangle }_{2}+{|1\rangle }_{1}{|-1\rangle }_{2}$$ (1)

which is the result of the conservation of the total OAM from the pump photons (ℓ = 0) to the signal and idler photons emitted by the SPDC process. Such a state will violate a Bell inequality of the form (*20*)

∣S∣≤2 (2)

with

$$S=E(\theta_{1},\theta_{2})-E(\theta_{1}^{\prime },\theta_{2})+E(\theta_{1},\theta_{2}^{\prime })+E(\theta_{1}^{\prime },\theta_{2}^{\prime })$$ (3)

and

$$E(\theta_{1},\theta_{2})=\frac{C(\theta_{1},\theta_{2})+C(\theta_{1}+\frac{\pi }{2},\theta_{2}+\frac{\pi }{2})-C(\theta_{1}+\frac{\pi }{2},\theta_{2})-C(\theta_{1},\theta_{2}+\frac{\pi }{2})}{C(\theta_{1},\theta_{2})+C(\theta_{1}+\frac{\pi }{2},\theta_{2}+\frac{\pi }{2})+C(\theta_{1}+\frac{\pi }{2},\theta_{2})+C(\theta_{1},\theta_{2}+\frac{\pi }{2})}$$ (4)

where *C*(θ_1_, θ_2_) is the recorded coincidence rate when the first photon is detected after a phase step with the orientation θ_1_ and when the second photon is measured after a phase step with the orientation θ_2_. The inequality (Eq. 2) is a Clauser-Horne-Shimony-Holt (CHSH) Bell inequality (*37*). As in a demonstration using the polarization degree of freedom, the state (Eq. 1) will exhibit a maximal violation of the inequality (Eq. 2) when the settings are chosen in the following way: θ_1_ = 22.5°, $\theta_{1}^{\prime }=67.5{}^{\circ}$, θ_2_ = 0°, and $\theta_{2}^{\prime }=45{}^{\circ}$. In our implementation, all the orientations θ_1_ in arm 1 necessary to perform the Bell test are obtained simply by using a two-dimensional circular phase step as the displayed object on SLM 1. As may be seen in Fig. 1, one needs to have four different orientations for the spatial phase step filter in the second arm (0°, 45°, 90°, 135°).

In our implementation, to perform imaging of the Bell inequality, we used the reduced state (Eq. 1) in conjugation with the spatial correlations exhibited by the EPR state generated through SPDC to acquire a spatially resolved image of the Bell behavior. We applied the phase filter in a Fourier plane of the crystal and placed the object in an image plane to ensure that the filtering effect will be applied to all the edges within the whole phase object plane, thus ensuring that simply taking a heralded ghost image of the object will give us access to many coincidence measurements in parallel across the ICCD camera, i.e., for the full 0 to 2π range of θ_2_ present in the object. Note that our intention here is not to target a loophole-free test. The detector efficiencies (∼10% for the ICCD camera and ∼50% for the SPAD) do not allow the closing of the detection loophole; moreover, the technical triggering process of the camera used here means that neither is the communication loophole closed in our implementation because a classical trigger signal is actually conveyed from one detector to the other.

Last, our demonstration does not ensure the randomization of the analyzer settings for both photons, which leads again to a loophole. In our experiment, that is based both on imaging and on a projection in the OAM basis, the random setting of the phase filter orientation does ensure a randomization of the basis for the detection of the second photon. However, the use of a fixed image in the other arm means that it is the different spatial positions in the image that correspond to the different orientations of the phase step. For this second process to be random, we need to assume that the position of generation of the photon pairs is also random and, more subtly, that this position is not linked, by some unknown process, to the OAM state of the light. Although both of these assumptions are reasonable in relation to our source of entangled photons, it is noteworthy that any claim of genuine nonlocal behavior depends on these assumptions. This caveat is the same for all demonstrations that are not loophole free, for example, a detection loophole requires a fair sampling assumption (*38*). However, it is also to be noted that in our case, these caveats are imposed by technical limitations rather than by fundamental limitations. For example, the way the phase object is displayed can be varied for each shot before being reconstructed to lead to a free choice of measurements performed on each side. A possible approach to implement this and to break the link between the lateral position of the photon and the corresponding angle of the edge of the phase circle is to apply a randomized scan of the lateral position of the phase circle and then, after measurement, to “de-scan” the associated component of the detected image. In the last part of Results, we report a successful implementation of these changes to the object displayed in arm 1. However, note that with the existing technology, such a scan cannot be made sufficiently fast to overcome the locality loophole.

Nevertheless, rather than targeting a fundamental loophole-free demonstration of nonlocality that has already been demonstrated (*5*–*7*), here we aim to demonstrate that it is possible to use a full-field imaging system and quantum imaging tools and techniques to reveal the Bell-type–violating behavior of a quantum system. This allows the Bell test to be performed in the context of high dimensionality and with a highly parallel measurement acquisition method.

### Bell inequality violation in four images

**Fig. 2:**
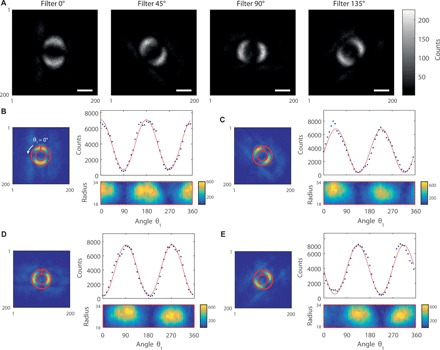
Full-frame images recording the violation of a Bell inequality in four images. (**A**) The four coincidence counting images are presented, which correspond to images of the phase circle acquired with the four phase filters with different orientations, θ_2_ = {0° , 45° , 90° , 135°}, necessary to perform the Bell test. Scale bars, 1 mm (in the plane of the object). (**B** to **E**) The coincidence counts graphs as a function of the orientation angle θ_1_ of the phase step along the object are presented. As shown, these results are obtained by unfolding the ROIs represented as red rings and are extracted from the images presented in (A). The blue dots in the graphs are the coincidence counts per angular region within the ROIs, and the red curves correspond to the best fits of the experimental data by a cosine-squared function. (B) to (E) correspond to phase filter orientations θ_2_ of 0°, 45°, 90°, and 135°, respectively.

In the first implementation of this experiment, we acquired four separate images corresponding to coincidence images of the ghost object filtered respectively by the four orientations, θ_2_ = {0° , 45° , 90° , 135° }, of the π-phase filter. The images obtained directly by summing the thresholded frames acquired by the ICCD camera are shown in Fig. 2A. As discussed before, these coincidence counting patterns are likely to include the signature of Bell-type behavior, and we can use these images to test the Bell inequality (Eq. 2). For this purpose, one can define a ring-like region of interest (ROI) along the edge of the phase circle object within each of these images, as shown in Fig. 2 (B to E). One can then unfold these ROIs by defining angular and radius bins within them and representing the images in polar coordinates. After integrating along the different radii within the ROIs, one obtains the graphs presented in Fig. 2 (B to E), which correspond respectively to the four orientations, θ_2_ = {0° , 45° , 90° , 135°}, of the π-phase filter. These graphs represent the coincidence counts as a function of the π-phase angle θ_1_ along the phase circle. As can be seen, the experimental data extracted from the images closely follow the expected sine-squared Malus-like behavior, and one can test the Bell inequality (Eq. 2) by selecting particular values of θ_1_ within these graphs. When selecting the angles such that θ_1_ = 22.5°, $\theta_{1}^{\prime }=67.5{}^{\circ}$, θ_2_ = 0°, and $\theta_{2}^{\prime }=45{}^{\circ}$, the Bell inequality is expected to be maximally violated.

By proceeding to such a Bell test, we find

S=2.4626±0.0261 (5)

that is, results demonstrating a Bell-type nonlocal behavior of the two-photon state and separated from a classical behavior (*S* ≤ 2) by more than 17 SDs.

Despite exceeding the classical limit of *S* > 2, the nonperfect contrast obtained on the graphs presented in Fig. 2 (B to E) explains that the ultimate two-dimensional $2\sqrt{2}$ bound for *S* is not saturated. This imperfect contrast arises from several factors. First, the nonperfect spatial coherence of the two-photon interference due to the finite size of the SMF core (*39*) can lead to lower contrast. Second, the camera technical noise together with the presence of parasitic light can further reduce the contrast. Last, the imperfect filtering of the phase circle by the phase filter can lead to a similar effect even for a perfectly coherent imaging scheme using an ideal detector. This latter effect has been evidenced by establishing some simulations that are reported in the Supplementary Materials.

Last, it is noteworthy that the images presented in Fig. 2A are the results of the remote interferometric filtering of the phase circle present in arm 1 by the phase step filter present in arm 2. Therein, the type of imaging performed here is more complex than conventional ghost imaging schemes. We see no easy way of qualitatively reproducing our imaging results using only classical correlations, let alone the quantitative violation of a Bell inequality that we report here, which requires entanglement.

### Bell inequality violation in a single image

**Fig. 3:**
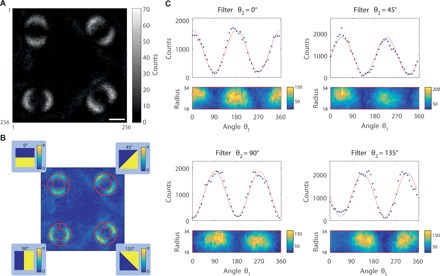
Full-frame single image recording the violation of a Bell inequality. (**A**) The coincidence counting single image acquired through our protocol is presented, which corresponds to an image of the same phase circle acquired with the four phase filters with different orientations, θ_2_ = {0° , 45° , 90° , 135°}, necessary to perform the Bell test. Scale bar, 1 mm (in the plane of the object). (**B**) The correspondence between the phase filters used and the particular observation of the object acquired in the single image are highlighted. The four ROIs used to treat the single image are also highlighted in (B). (**C**) The coincidence counts graphs as a function of the orientation angle θ_1_ of the phase step along the object for the four different orientations of the phase filters are presented. These graphs are obtained solely by extracting the coincidence counts in the single image presented in (A).

In a second implementation of the experiment, we perform a demonstration of a violation of a Bell-type inequality within a single accumulated image to demonstrate the capability of quantum imaging to access highly dimensional parallel measurements. To observe the single phase circle object as filtered by the four different phase filters in a single image acquired by the camera, we add to the phase filters displayed on SLM 2 a different blazed grating for each orientation θ_2_ of the phase filters’ phase step. In this way, we deviate the beam in arm 2 in a different manner for each filter and, therefore, acquire four concurrent images of the phase circle in different parts of the photosensitive array of the camera. During the exposure time of each frame acquired by the camera, we randomly choose the phase mask displayed on SLM 2 to switch in between the four different phase filters θ_2_ = {0° , 45° , 90° , 135° } randomly and with equal probability. One can then accumulate the single image shown in Fig. 3A, and through a similar treatment of the images as before, defining the four ROIs shown in Fig. 3B, one obtains the curves in Fig. 3C expressing the coincidence counts as a function of θ_1_ for the four different phase filter orientations θ_2_.

Again, one can use these data extracted from the single image to perform a test of the Bell inequality (Eq. 2). By using the following set of angles, θ_1_ = 22.5°, θ_1^′^_ = 67.5°, θ_2_ = 0°, and $\theta_{2}^{\prime }=45{}^{\circ}$, one find

S=2.443±0.038 (6)

that is, demonstrating a Bell-type nonlocal behavior in the single image. The results are, in that latter case, separated from classical behavior by more than 11 SDs.

### Experimental realization with time-varying displacement of the phase object

**Fig. 4:**
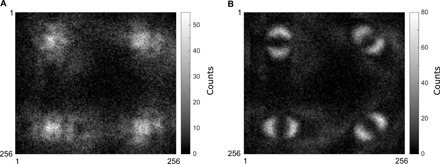
Full-frame single image recording the violation of a Bell inequality and implementing the scanning of the phase circle. (**A**) The raw sum of the coincidence counting single image acquired through our protocol is presented, which corresponds to an image of the same phase circle acquired with the four phase filters with different orientations, θ_2_ = {0° , 45° , 90° , 135° }, necessary to perform the Bell test. (**B**) The image obtained by de-scanning each of the images given the chosen position for the phase circle is presented. We can use this latter image to perform an evaluation of the Bell parameter *S* and to demonstrate the nonlocal behavior.

To be able to close one of the existing loopholes in our demonstration, we can introduce a time-varying displacement of the phase circle displayed on SLM 1 (in arm 1 of the setup) and apply a corresponding de-scan to the photon detection on the ICCD camera. To keep the moving circle within the field of view in arm 1, we slightly reduced its size to a radius of 21 pixels. The circle is moved between four different possible positions around the center of the beam. Taking the center of the beam as origin (0,0), the four possible positions in numbers of pixels are (10,10), (10,−10), (−10,10), and (−10,−10). We then reproduce the same acquisition of single images as presented previously, with the difference now being that, for each of the images, a position of the phase circle is chosen, and we keep track of this position. A raw sum of the images thus acquired is presented in Fig. 4A. One can observe that we have still four different parts in the image, each corresponding to the different orientations of the phase filter in arm 2, but the expected filtered phase circles do not appear anymore because of the scanning of the phase circle to different transverse positions. However, one can then use the information of the position of the phase circle to de-scan each of the images and then again summing all of the images together. The result is shown in Fig. 4B, where one can see once again the four distinctive filtered phase circles indicative of a test of a Bell inequality. One can now use this image in the same way as described previously to perform an evaluation of the Bell parameter. We find

S=2.183±0.084 (7)

that is, demonstrating a Bell-type nonlocal behavior in the single image. The results, are in that latter case, separated from classical behavior by more than 2.17 SDs.

## DISCUSSION

In this work, we proposed and demonstrated the use of an imaging scheme to perform a demonstration of a Bell-type inequality. We tested the Bell inequality here in two dimensions through a conventional CHSH inequality but exploited the high dimensionality of the transverse spatial variables to obtain a test performed and apparent in a single accumulated image. Nevertheless, note that one could use a similar setup to attain higher dimensions in which the Bell inequality is tested by using more complex holograms involving higher-order OAMs (*21*). A trade-off between the resolution of the acquired images and the dimensionality in which a Bell inequality is tested is then expected to occur. As discussed, our demonstration is not exempt of loopholes, but these loopholes could potentially be technically addressed and closed. Moreover, our demonstration shows that one can detect the signature of a Bell-type behavior within a single image acquired by an imaging setup. By demonstrating that quantum imaging can generate high-dimensional images illustrating the presence of Bell-type entanglement, we benchmark quantum imaging techniques against the most fundamental test of quantum mechanics. Such a benchmark generally holds a unique status in that it is a good way to assess whether and how well quantum protocols will perform within a given system. Our demonstration therefore suggests that quantum imaging techniques can benefit from any advantages provided by the use of quantum illumination. In particular, this result both opens the way to new quantum imaging schemes based on the violation of a Bell inequality and constitutes a demonstration that the transverse spatial variables of light can be exploited to enable highly parallel acquisitions of fundamental quantum features, suggesting promise for quantum information schemes based on spatial variables. We hope that the present work will inspire and generate a new class of quantum imaging demonstrations and techniques relying on Bell-type entanglement to extract imaging advantages out of quantum-correlated sources that could not be obtained classically.

## MATERIALS AND METHODS

The four images shown in Fig. 3A and the single image shown in Fig. 2A were each obtained by acquiring 40,000 frames each of 1 s of exposure, during which time the camera intensifier was triggered for every heralding detection by the SPAD. The ICCD sensor was air cooled to −30°C. The images were thresholded to generate binary images that correspond to the detection of single photons. We calculated the threshold over which a pixel was considered to correspond to a photo-detection and the noise probability per pixel by acquiring 5000 frames with the camera optical input blocked. The dark count probability per pixel and per frame arising from the camera readout noise was then calculated to be around 5 × 10^−5^.

The images obtained correspond to photon correlation images, the intensity in the images corresponds to the number of coincidence counts because the camera is triggered by the detection of the first photon by the SPAD, and the images are then analyzed to test the Bell inequality. First, we located the center of each image circle, and we defined ring-like ROIs to follow the edges of each object. These rings are 17 pixels in width with a mean radius of 26 pixels. The coincidence counting images within the ROIs were then converted into polar coordinates. We used 48 angular bins from 0 to 2π, and we integrated over the 17-pixel width of the ROIs to obtain the coincidence as a function of the angle θ_1_ corresponding to the local orientation of the π-phase step at a particular position on the camera. From these data points, one can read the coincidence rates corresponding to the angles of interest to perform the Bell test.

Uncertainties on the mean value of *S* were obtained as SEs by splitting the set of 40,000 frames into 20 parts of 2000 frames and evaluating for each of the 20 sets a value for *S*. With these 20 values of *S*, we then computed the means and the SEM. Note that a detailed schematic of the experimental setup is available in the Supplementary Materials.

## Supplementary Material

http://advances.sciencemag.org/cgi/content/full/5/7/eaaw2563/DC1

Download PDF

## SUPPLEMENTARY MATERIALS

Supplementary material for this article is available at http://advances.sciencemag.org/cgi/content/full/5/7/eaaw2563/DC1 Section S1. Detailed experimental setup Section S2. Classical simulations Fig. S1. Detailed experimental setup. Fig. S2. Simulated image for realistic parameters. Reference (*40*)

## References

[1] Freedman S. J., Clauser J. F., Experimental test of local hidden-variable theories. Phys. Rev. Lett. 28, 938–941 (1972).

[2] Aspect A., Grangier P., Roger G., Experimental tests of realistic local theories via Bell’s theorem. Phys. Rev. Lett. 47, 460–463 (1981).

[3] Aspect A., Grangier P., Roger G., Experimental realization of Einstein-Podolsky-Rosen-Bohm *Gedankenexperiment*: A new violation of Bell’s inequalities. Phys. Rev. Lett. 49, 91–94 (1982).

[4] Aspect A., Dalibard J., Roger G., Experimental test of Bell’s inequalities using time-varying analyzers. Phys. Rev. Lett. 49, 1804–1807 (1982).

[5] Hensen B., Bernien H., Dréau A. E., Reiserer A., Kalb N., Blok M. S., Ruitenberg J., Vermeulen R. F. L., Schouten R. N., Abellán C., Amaya W., Pruneri V., Mitchell M. W., Markham M., Twitchen D. J., Elkouss D., Wehner S., Taminiau T. H., Hanson R., Loophole-free bell inequality violation using electron spins separated by 1.3 kilometres. Nature 526, 682–686 (2015). 26503041 10.1038/nature15759

[6] Giustina M., Versteegh M. A. M., Wengerowsky S., Handsteiner J., Hochrainer A., Phelan K., Steinlechner F., Kofler J., Larsson J.-Å., Abellán C., Amaya W., Pruneri V., Mitchell M. W., Beyer J., Gerrits T., Lita A. E., Shalm L. K., Nam S. W., Scheidl T., Ursin R., Wittmann B., Zeilinger A., Significant-loophole-free test of Bell’s theorem with entangled photons. Phys. Rev. Lett. 115, 250401 (2015). 26722905 10.1103/PhysRevLett.115.250401

[7] Shalm L. K., Meyer-Scott E., Christensen B. G., Bierhorst P., Wayne M. A., Stevens M. J., Gerrits T., Glancy S., Hamel D. R., Allman M. S., Coakley K. J., Dyer S. D., Hodge C., Lita A. E., Verma V. B., Lambrocco C., Tortorici E., Migdall A. L., Zhang Y., Kumor D. R., Farr W. H., Marsili F., Shaw M. D., Stern J. A., Abellán C., Amaya W., Pruneri V., Jennewein T., Mitchell M. W., Kwiat P. G., Bienfang J. C., Mirin R. P., Knill E., Nam S. W., Strong loophole-free test of local realism. Phys. Rev. Lett. 115, 250402 (2015). 26722906 10.1103/PhysRevLett.115.250402 PMC5815856

[8] Acín A., Brunner N., Gisin N., Massar S., Pironio S., Scarani V., Device-independent security of quantum cryptography against collective attacks. Phys. Rev. Lett. 98, 230501 (2007). 17677888 10.1103/PhysRevLett.98.230501

[9] Pironio S., Acín A., Massar S., de la Giroday A. B., Matsukevich D. N., Maunz P., Olmschenk S., Hayes D., Luo L., Manning T. A., Monroe C., Random numbers certified by Bell’s theorem. Nature 464, 1021–1024 (2010). 20393558 10.1038/nature09008

[10] Šupić I., Augusiak R., Salavrakos A., Acín A., Self-testing protocols based on the chained Bell inequalities. New J. Phys. 18, 035013 (2016).

[11] Rarity J. G., Tapster P. R., Experimental violation of Bell’s inequality based on phase and momentum. Phys. Rev. Lett. 64, 2495–2498 (1990). 10041727 10.1103/PhysRevLett.64.2495

[12] Reid M. D., Walls D. F., Violations of classical inequalities in quantum optics. Phys. Rev. A 34, 1260–1276 (1986). 10.1103/physreva.34.1260 9897387

[13] Guo X., Mei Y., Du S., Testing the bell inequality on frequency-bin entangled photon pairs using time-resolved detection. Optica 4, 388–392 (2017).

[14] The BIG Bell Test Collaboration, Challenging local realism with human choices. Nature 557, 212–216 (2018). 29743691 10.1038/s41586-018-0085-3

[15] Collins D., Gisin N., Linden N., Massar S., Popescu S., Bell inequalities for arbitrarily high-dimensional systems. Phys. Rev. Lett. 88, 040404 (2002). 11801097 10.1103/PhysRevLett.88.040404

[16] Allen L., Beijersbergen M. W., Spreeuw R. J. C., Woerdman J. P., Orbital angular momentum of light and the transformation of Laguerre-Gaussian laser modes. Phys. Rev. A 45, 8185–8189 (1992). 9906912 10.1103/physreva.45.8185

[17] L. Allen, S. M. Barnett, M. J. Padgett, *Optical Angular Momentum* (CRC Press, 2003).

[18] He H., Friese M. E. J., Heckenberg N. R., Rubinsztein-Dunlop H., Direct observation of transfer of angular momentum to absorptive particles from a laser beam with a phase singularity. Phys. Rev. Lett. 75, 826–829 (1995). 10060128 10.1103/PhysRevLett.75.826

[19] Mair A., Vaziri A., Weihs G., Zeilinger A., Entanglement of the orbital angular momentum states of photons. Nature 412, 313–316 (2001). 11460157 10.1038/35085529

[20] Leach J., Jack B., Romero J., Ritsch-Marte M., Boyd R. W., Jha A. K., Barnett S., Franke-Arnold S., Padgett M. J., Violation of a bell inequality in two-dimensional orbital angular momentum state-spaces. Opt. Express 17, 8287–8293 (2009). 19434161 10.1364/oe.17.008287

[21] Dada A. C., Leach J., Buller G. S., Padgett M. J., Andersson E., Experimental high-dimensional two-photon entanglement and violations of generalized Bell inequalities. Nat. Phys. 7, 677–680 (2011).

[22] Einstein A., Podolsky B., Rosen N., Can quantum-mechanical description of physical reality be considered complete? Phys. Rev. 47, 777–780 (1935).

[23] Pittman T. B., Shih Y. H., Strekalov D. V., Sergienko A. V., Optical imaging by means of two-photon quantum entanglement. Phys. Rev. A 52, R3429–R3432 (1995). 9912767 10.1103/physreva.52.r3429

[24] Moreau P.-A., Toninelli E., Gregory T., Padgett M. J., Ghost imaging using optical correlations. Laser Photonics Rev. 12, 1700143 (2018).

[25] Aspden R. S., Tasca D. S., Boyd R. W., Padgett M. J., EPR-based ghost imaging using a single-photon-sensitive camera. New J. Phys. 15, 073032 (2013).

[26] Devaux F., Mougin-Sisini J., Moreau P.-A., Lantz E., Towards the evidence of a purely spatial Einstein-Podolsky-Rosen paradox in images: Measurement scheme and first experimental results. Eur. Phys. J. B 66, 192 (2012).

[27] Moreau P.-A., Mougin-Sisini J., Devaux F., Lantz E., Realization of the purely spatial Einstein-Podolsky-Rosen paradox in full-field images of spontaneous parametric down-conversion. Phys. Rev. A 86, 010101 (2012).

[28] Edgar M. P., Tasca D. S., Izdebski F., Warburton R. E., Leach J., Agnew M., Buller G. S., Boyd R. W., Padgett M. J., Imaging high-dimensional spatial entanglement with a camera. Nat. Commun. 3, 984 (2012). 22871804 10.1038/ncomms1988 PMC3432466

[29] Moreau P.-A., Devaux F., Lantz E., Einstein-Podolsky-Rosen paradox in twin images. Phys. Rev. Lett. 113, 160401 (2014). 25361237 10.1103/PhysRevLett.113.160401

[30] Lantz E., Denis S., Moreau P.-A., Devaux F., Einstein-Podolsky-Rosen paradox in single pairs of images. Opt. Express 23, 26472–26478 (2015). 26480160 10.1364/OE.23.026472

[31] Jack B., Leach J., Romero J., Franke-Arnold S., Ritsch-Marte M., Barnett S. M., Padgett M. J., Holographic ghost imaging and the violation of a bell inequality. Phys. Rev. Lett. 103, 083602 (2009). 19792729 10.1103/PhysRevLett.103.083602

[32] Morris P. A., Aspden R. S., Bell J. E. C., Boyd R. W., Padgett M. J., Imaging with a small number of photons. Nat. Commun. 6, 5913 (2015). 25557090 10.1038/ncomms6913 PMC4354036

[33] T. Aidukas, P. C. Konda, A. R. Harvey, M. J. Padgett, P.-A. Moreau, Phase and amplitude imaging with quantum correlations through Fourier ptychography. arXiv:1906.06569 (2019). 10.1038/s41598-019-46273-x PMC6639395 31320691

[34] Moreau P.-A., Toninelli E., Morris P. A., Aspden R. S., Gregory T., Spalding G., Boyd R. W., Padgett M. J., Resolution limits of quantum ghost imaging. Opt. Express 26, 7528–7536 (2018). 29609307 10.1364/OE.26.007528

[35] Moreau P.-A., Morris P. A., Toninelli E., Gregory T., Aspden R. S., Spalding G., Boyd R. W., Padgett M. J., Experimental limits of ghost diffraction: Popper’s thought experiment. Sci. Rep. 8, 13183 (2018). 30181599 10.1038/s41598-018-31429-y PMC6123420

[36] Padgett M. J., Courtial J., Poincaré-sphere equivalent for light beams containing orbital angular momentum. Opt. Lett. 24, 430–432 (1999). 18071529 10.1364/ol.24.000430

[37] Clauser J. F., Horne M. A., Shimony A., Holt R. A., Proposed experiment to test local hidden-variable theories. Phys. Rev. Lett. 23, 880–884 (1969).

[38] Brunner N., Cavalcanti D., Pironio S., Scarani V., Wehner S., Bell nonlocality. Rev. Mod. Phys. 86, 419–478 (2014).

[39] Tasca D. S., Aspden R. S., Morris P. A., Anderson G., Boyd R. W., Padgett M. J., The influence of non-imaging detector design on heralded ghost-imaging and ghost-diffraction examined using a triggered ICCD camera. Opt. Express 21, 30460–30473 (2013). 24514623 10.1364/OE.21.030460

[40] Klyshko D., A simple method of preparing pure states of the optical-field, a realization of the Einstein, Podolsky, Rosen experiment and a demonstration of the complementarity principle. Usp. Fiz. Nauk 154, 133–152 (1988).

