# Supplementary material for: Imaging Bell-type nonlocal behavior
Source: Sci Adv. 2019 Jul 12;5(7):eaaw2563. doi: 10.1126/sciadv.aaw2563 (PMC6625815; doi:10.1126/sciadv.aaw2563)
Supplement: http://advances.sciencemag.org/cgi/content/full/5/7/eaaw2563/DC1 [file supp_5_7_eaaw2563__index.html]

Science Advances | Science AdvancesAAASSearchScience AdvancesMenu

## Supplementary Materials

**This PDF file includes:**

- Section S1. Detailed experimental setup
- Section S2. Classical simulations
- Fig. S1. Detailed experimental setup.
- Fig. S2. Simulated image for realistic parameters.
- Reference (*40*)

Download PDF

**Files in this Data Supplement:**

- Adobe PDF - aaw2563\_SM.pdf
