# Supplementary material for: Imaging Bell-type nonlocal behavior
Source: Sci Adv. 2019 Jul 12;5(7):eaaw2563. doi: 10.1126/sciadv.aaw2563 (PMC6625815; doi:10.1126/sciadv.aaw2563)
Supplement: Download PDF [file aaw2563_SM.pdf]

Science Advances
AAAS

advances.sciencemag.org/cgi/content/full/5/7/eaaw2563/DC1

Supplementary Materials for

Imaging Bell-type nonlocal behavior

Paul-Antoine Moreau*, Ermes Toninelli, Thomas Gregory, Reuben S. Aspden, Peter A. Morris, Miles J. Padgett*

*Corresponding author. Email: paul-antoine.moreau@glasgow.ac.uk (P.-A.M.); miles.padgett@glasgow.ac.uk (M.J.P.)

Published 12 July 2019, *Sci. Adv.* **5**, eaaw2563 (2019)
DOI: 10.1126/sciadv.aaw2563

This PDF file includes:

Section S1. Detailed experimental setup
Section S2. Classical simulations
Fig. S1. Detailed experimental setup.
Fig. S2. Simulated image for realistic parameters.
Reference (*40*)

Section S1. Detailed experimental setup

A detailed version of the experimental setup is shown in Fig. 1. All lenses are placed in a f-f configuration (i.e. each couple of successive lenses forms a 4f system), with the exception of the lens between SLM 1 and the fiber coupler which is in a 2f-2f configuration, thus re-imaging SLM 1 around the object focal plane of the fiber coupler lens. As can be seen in Fig. 1 the delay line is composed of an optical isolator implemented with a polarising beam-splitter (PBS) and a quarter wave plate (QWP).

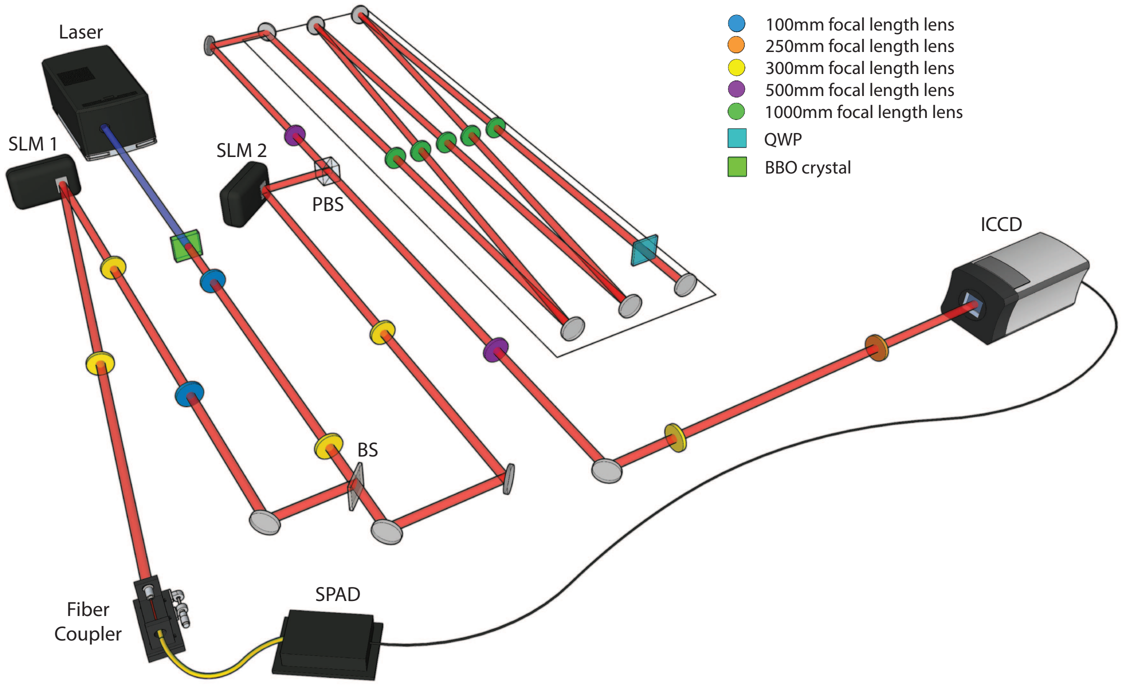

Fig. S1. **Detailed experimental setup.**

Section S2. Classical simulations

We have written classical simulations based on the Klyshko advanced wave picture [40]. This picture tells us that the two photon coincidence rate can be simulated by replacing one of the two detectors with an optical source with the same spatial properties. The light originating from this source is retro-propagated through the same optical system to the crystal, upon which the photon is reflected, and is then propagated through the other arm up to the second detector. Applying such a picture, the setup presented in Fig. 1 becomes simply an imaging system in which the object displayed on SLM 1 is illuminated with a coherent source and is filtered by the spatial filter displayed on SLM 2 before being imaged onto the ICCD camera. We have therefore simply simulated such a Fourier-filtering imaging scheme with the object and phase filter involved in our demonstration. Different parameters have been introduced to match the experimental conditions: The size of the object; resolution of the optical system, which is set by the spatial extent of the correlations between the plane of the camera and the plane of SLM 1 (measured experimentally); spatial extent of the SPDC beam in the plane of SLM 1 (measured experimentally).

In figure 2a we show an image simulated under realistic conditions but with no simulation of the non-ideal nature of the detector. It can be seen on the simulated image that the filtering effect of the spatial filter is not perfect and that some of the filtered out light that appears to be rejected outside of the ring is actually not rejected to a great enough extent and therefore results in a parasitic background within the desired interference pattern. One finds a background of around 3% of the maximal value, on the simulated 'coincidence count' graph as a function of the angle θ_1 (Fig. 2b) extracted from the images in the same manner as explained in the main text. By producing four images for the four orientations of the phase filter one can extract a classically simulated value for $S = 2.6607$.

Therefore, without considering the non-ideal nature of the detector, the presence of this background, which is a result of the filtering effect, in the interference pattern already prevents us from saturating the bound of maximal violation of the CHSH Bell inequality.
This is in addition to the non-ideal nature of the experiment in which the technical noise of the camera and non-prefect visibility of the interference pattern due to the non-perfect spatial coherence of the beams further restrict our attempts to saturate this bound.

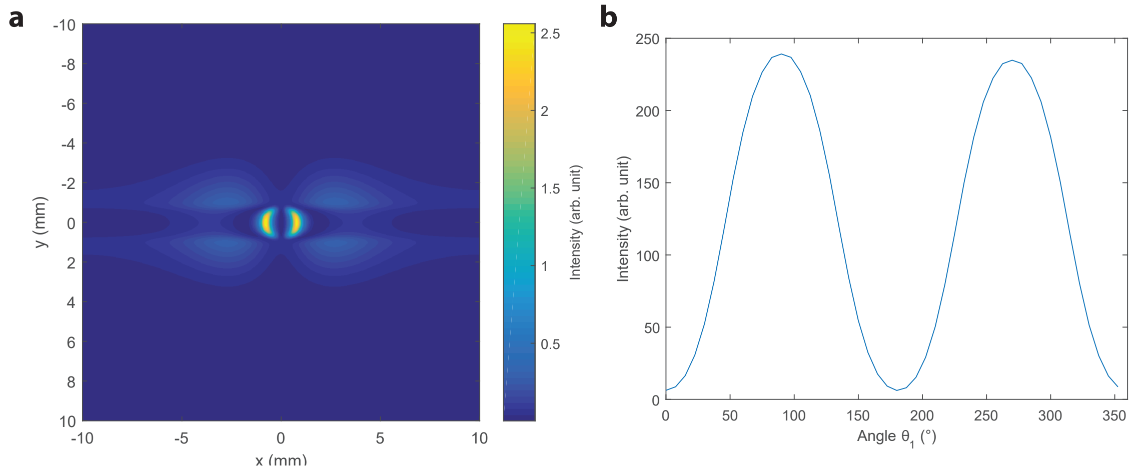

Fig. S2. **Simulated image for realistic parameters**. The image is obtained here for a phase filter oriented at 90°. **a** is the simulated image. **b** is the intensity distribution as a function of the angle θ_1 of the phase step around the circle. This curve simulates the two photon coincidence rate as a function of θ_1 obtained experimentally.
